# 2-(7-Methyl-1*H*-indol-3-yl)acetonitrile

**DOI:** 10.1107/S1600536811053396

**Published:** 2011-12-17

**Authors:** Yu-Hua Ge, Mei-Ling Pan, Jian Xu, Yang-Hui Luo

**Affiliations:** a Ordered Matter Science Research Center, College of Chemistry and Chemical Engineering, Southeast University, Nanjing 210096, People’s Republic of China

## Abstract

In the title compound, C_11_H_10_N_2_, the carbonitrile group is twisted away from the indole plane [C_cy_—C_me_—C_ar_—C_ar_ = 66.6 (2)°; cy = cyanide, me = methyl­ene and ar = aromatic]. In the crystal, N—H⋯N hydrogen bonds link the mol­ecules into *C*(7) chains propagating in the [001] direction.

## Related literature

For background to indole derivatives as pharmaceuticals, see: Kunzer & Wendt (2011 ▶).
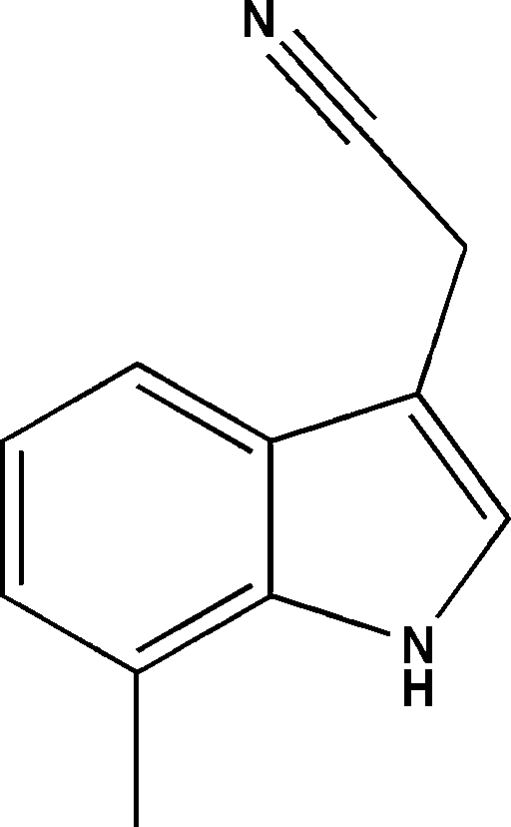


## Experimental

### 

#### Crystal data

C_11_H_10_N_2_
*M* *_r_* = 170.21
Monoclinic, 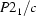

*a* = 6.9962 (14) Å
*b* = 8.9445 (18) Å
*c* = 15.406 (3) Å
β = 97.97 (3)°
*V* = 954.7 (3) Å^3^
*Z* = 4
Mo *K*α radiation
μ = 0.07 mm^−1^
*T* = 293 K
0.26 × 0.24 × 0.15 mm

#### Data collection

Rigaku SCXmini CCD diffractometer
Absorption correction: multi-scan (*CrystalClear*; Rigaku, 2005 ▶) *T*_min_ = 0.981, *T*_max_ = 0.989
9402 measured reflections
2160 independent reflections
1418 reflections with *I* > 2σ(*I*)
*R*_int_ = 0.058

#### Refinement

*R*[*F* ^2^ > 2σ(*F* ^2^)] = 0.055
*wR*(*F* ^2^) = 0.154
*S* = 1.05
2160 reflections
118 parameters
H-atom parameters constrained
Δρ_max_ = 0.16 e Å^−3^
Δρ_min_ = −0.17 e Å^−3^

### 

Data collection: *CrystalClear* (Rigaku, 2005 ▶); cell refinement: *CrystalClear*; data reduction: *CrystalClear*; program(s) used to solve structure: *SHELXS97* (Sheldrick, 2008 ▶); program(s) used to refine structure: *SHELXL97* (Sheldrick, 2008 ▶); molecular graphics: *DIAMOND* (Brandenburg & Putz, 2005 ▶); software used to prepare material for publication: *SHELXL97*.

## Supplementary Material

Crystal structure: contains datablock(s) I, global. DOI: 10.1107/S1600536811053396/hb6546sup1.cif

Structure factors: contains datablock(s) I. DOI: 10.1107/S1600536811053396/hb6546Isup2.hkl

Supplementary material file. DOI: 10.1107/S1600536811053396/hb6546Isup3.cml

Additional supplementary materials:  crystallographic information; 3D view; checkCIF report

## Figures and Tables

**Table 1:** Hydrogen-bond geometry (Å, °)

*D*—H⋯*A*	*D*—H	H⋯*A*	*D*⋯*A*	*D*—H⋯*A*
N1—H1*A*⋯N2^i^	0.86	2.24	3.022 (2)	151

Symmetry code: (i) 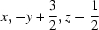.

**Fig. 1.**
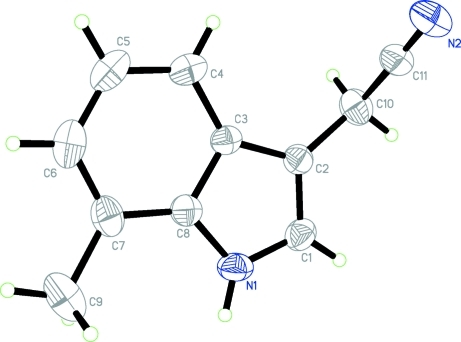
The molecular structure of the title compound with displacement ellipsoids drawn at the 30% probability level.

**Fig. 2.**
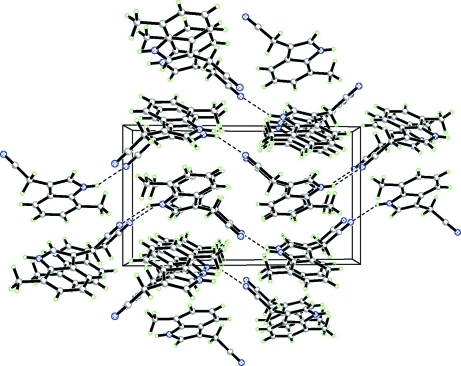
A packing view down the a axis with hydrogen bonds shown as dashed lines.

## supplementary crystallographic information

### Experimental

Colourless blocks of (I) were obstained by slow evaporation of a methanol solution of a commercially supplied sample.

### Refinement

All H atoms attached to C atoms and O atoms were fixed geometrically and treated as riding with C—H = 0.93 Å (CH), C—H = 0.97 Å (CH_2_), C—H = 0.96 Å (CH_3_)and N—H = 0.86 Å with *U*_iso_(H) = 1.2*U*_eq_(CH, CH_2_ and NH) and *U*_iso_(H) = 1.5*U*_eq_(CH_3_).

### Crystal data

**Table:**

C_11_H_10_N_2_	*F*(000) = 360
*M**_r_* = 170.21	*D*_x_ = 1.184 Mg m^−^^3^
Monoclinic, *P*2_1_/*c*	Mo *K*α radiation, λ = 0.71073 Å
Hall symbol: -P 2ybc	Cell parameters from 2175 reflections
*a* = 6.9962 (14) Å	θ = 2.7–27.5°
*b* = 8.9445 (18) Å	µ = 0.07 mm^−^^1^
*c* = 15.406 (3) Å	*T* = 293 K
β = 97.97 (3)°	Block, colourless
*V* = 954.7 (3) Å^3^	0.26 × 0.24 × 0.15 mm
*Z* = 4	

### Data collection

**Table:**

Rigaku SCXmini CCD diffractometer	2160 independent reflections
Radiation source: fine-focus sealed tube	1418 reflections with *I* > 2σ(*I*)
graphite	*R*_int_ = 0.058
ω scans	θ_max_ = 27.5°, θ_min_ = 3.5°
Absorption correction: multi-scan (*CrystalClear*; Rigaku, 2005)	*h* = −9→9
*T*_min_ = 0.981, *T*_max_ = 0.989	*k* = −11→11
9402 measured reflections	*l* = −19→19

### Refinement

**Table:**

Refinement on *F*^2^	Primary atom site location: structure-invariant direct methods
Least-squares matrix: full	Secondary atom site location: difference Fourier map
*R*[*F*^2^ > 2σ(*F*^2^)] = 0.055	Hydrogen site location: inferred from neighbouring sites
*wR*(*F*^2^) = 0.154	H-atom parameters constrained
*S* = 1.05	*w* = 1/[σ^2^(*F*_o_^2^) + (0.0708*P*)^2^ + 0.0707*P*] where *P* = (*F*_o_^2^ + 2*F*_c_^2^)/3
2160 reflections	(Δ/σ)_max_ < 0.001
118 parameters	Δρ_max_ = 0.16 e Å^−^^3^
0 restraints	Δρ_min_ = −0.17 e Å^−^^3^

### Special details

**Table:**

Geometry. All e.s.d.'s (except the e.s.d. in the dihedral angle between two l.s. planes) are estimated using the full covariance matrix. The cell e.s.d.'s are taken into account individually in the estimation of e.s.d.'s in distances, angles and torsion angles; correlations between e.s.d.'s in cell parameters are only used when they are defined by crystal symmetry. An approximate (isotropic) treatment of cell e.s.d.'s is used for estimating e.s.d.'s involving l.s. planes.
Refinement. Refinement of *F*^2^ against ALL reflections. The weighted *R*-factor *wR* and goodness of fit *S* are based on *F*^2^, conventional *R*-factors *R* are based on *F*, with *F* set to zero for negative *F*^2^. The threshold expression of *F*^2^ > σ(*F*^2^) is used only for calculating *R*-factors(gt) *etc*. and is not relevant to the choice of reflections for refinement. *R*-factors based on *F*^2^ are statistically about twice as large as those based on *F*, and *R*- factors based on ALL data will be even larger.

### Fractional atomic coordinates and isotropic or equivalent isotropic displacement parameters (Å^2^)

**Table:**

	*x*	*y*	*z*	*U*_iso_*/*U*_eq_	
C1	−0.0179 (3)	0.6121 (2)	0.21424 (12)	0.0587 (5)	
H1B	−0.1278	0.6642	0.1903	0.070*	
C2	0.0270 (2)	0.57655 (18)	0.30000 (11)	0.0511 (4)	
C3	0.2072 (2)	0.49843 (17)	0.30912 (10)	0.0473 (4)	
C4	0.3276 (3)	0.43296 (19)	0.37953 (12)	0.0613 (5)	
H4A	0.2955	0.4369	0.4361	0.074*	
C5	0.4936 (3)	0.3631 (2)	0.36311 (16)	0.0758 (6)	
H5A	0.5736	0.3187	0.4092	0.091*	
C6	0.5451 (3)	0.3571 (2)	0.27861 (17)	0.0768 (6)	
H6A	0.6588	0.3088	0.2703	0.092*	
C7	0.4334 (3)	0.4204 (2)	0.20730 (13)	0.0644 (5)	
C8	0.2629 (2)	0.48979 (17)	0.22467 (10)	0.0506 (4)	
C9	0.4896 (4)	0.4170 (3)	0.11583 (16)	0.0958 (8)	
H9A	0.6091	0.3639	0.1168	0.144*	
H9B	0.5047	0.5175	0.0959	0.144*	
H9C	0.3905	0.3676	0.0768	0.144*	
C10	−0.0948 (3)	0.6089 (2)	0.37116 (13)	0.0678 (5)	
H10A	−0.2171	0.6509	0.3449	0.081*	
H10B	−0.1216	0.5158	0.3994	0.081*	
C11	−0.0021 (3)	0.7128 (2)	0.43771 (12)	0.0621 (5)	
N1	0.1212 (2)	0.56067 (16)	0.16799 (9)	0.0600 (4)	
H1A	0.1207	0.5707	0.1124	0.072*	
N2	0.0698 (3)	0.7913 (2)	0.48993 (11)	0.0856 (6)	

### Atomic displacement parameters (Å^2^)

**Table:**

	*U*^11^	*U*^22^	*U*^33^	*U*^12^	*U*^13^	*U*^23^
C1	0.0586 (10)	0.0557 (10)	0.0580 (11)	0.0065 (8)	−0.0048 (9)	−0.0008 (8)
C2	0.0529 (9)	0.0500 (9)	0.0495 (10)	0.0010 (7)	0.0040 (7)	−0.0038 (7)
C3	0.0537 (9)	0.0413 (8)	0.0454 (9)	−0.0049 (7)	0.0021 (7)	0.0014 (6)
C4	0.0659 (11)	0.0584 (11)	0.0562 (10)	−0.0058 (9)	−0.0030 (9)	0.0094 (8)
C5	0.0656 (12)	0.0597 (12)	0.0951 (16)	0.0036 (10)	−0.0131 (11)	0.0164 (11)
C6	0.0601 (12)	0.0574 (12)	0.1126 (18)	0.0064 (9)	0.0108 (12)	−0.0067 (12)
C7	0.0642 (11)	0.0542 (11)	0.0780 (13)	−0.0045 (9)	0.0207 (10)	−0.0131 (9)
C8	0.0585 (10)	0.0425 (9)	0.0503 (9)	−0.0045 (7)	0.0055 (8)	−0.0040 (7)
C9	0.0977 (17)	0.0985 (17)	0.1011 (18)	−0.0132 (14)	0.0481 (14)	−0.0277 (13)
C10	0.0606 (11)	0.0731 (12)	0.0715 (12)	−0.0027 (9)	0.0156 (10)	−0.0097 (10)
C11	0.0675 (11)	0.0716 (12)	0.0490 (10)	0.0085 (10)	0.0150 (9)	0.0013 (9)
N1	0.0748 (10)	0.0628 (9)	0.0404 (8)	−0.0020 (8)	0.0012 (7)	0.0008 (6)
N2	0.1026 (14)	0.0986 (14)	0.0549 (10)	0.0050 (11)	0.0087 (9)	−0.0152 (9)

### Geometric parameters (Å, °)

**Table:**

C1—C2	1.353 (2)	C6—H6A	0.9300
C1—N1	1.364 (2)	C7—C8	1.403 (2)
C1—H1B	0.9300	C7—C9	1.515 (3)
C2—C3	1.431 (2)	C8—N1	1.381 (2)
C2—C10	1.507 (2)	C9—H9A	0.9600
C3—C4	1.405 (2)	C9—H9B	0.9600
C3—C8	1.411 (2)	C9—H9C	0.9600
C4—C5	1.373 (3)	C10—C11	1.467 (3)
C4—H4A	0.9300	C10—H10A	0.9700
C5—C6	1.399 (3)	C10—H10B	0.9700
C5—H5A	0.9300	C11—N2	1.131 (2)
C6—C7	1.378 (3)	N1—H1A	0.8600
			
C2—C1—N1	110.17 (15)	C8—C7—C9	121.5 (2)
C2—C1—H1B	124.9	N1—C8—C7	129.60 (17)
N1—C1—H1B	124.9	N1—C8—C3	106.97 (15)
C1—C2—C3	107.04 (15)	C7—C8—C3	123.43 (17)
C1—C2—C10	125.94 (16)	C7—C9—H9A	109.5
C3—C2—C10	126.99 (15)	C7—C9—H9B	109.5
C4—C3—C8	118.43 (16)	H9A—C9—H9B	109.5
C4—C3—C2	134.80 (16)	C7—C9—H9C	109.5
C8—C3—C2	106.76 (14)	H9A—C9—H9C	109.5
C5—C4—C3	118.63 (18)	H9B—C9—H9C	109.5
C5—C4—H4A	120.7	C11—C10—C2	112.97 (15)
C3—C4—H4A	120.7	C11—C10—H10A	109.0
C4—C5—C6	121.50 (19)	C2—C10—H10A	109.0
C4—C5—H5A	119.2	C11—C10—H10B	109.0
C6—C5—H5A	119.2	C2—C10—H10B	109.0
C7—C6—C5	122.37 (19)	H10A—C10—H10B	107.8
C7—C6—H6A	118.8	N2—C11—C10	179.0 (2)
C5—C6—H6A	118.8	C1—N1—C8	109.06 (14)
C6—C7—C8	115.63 (18)	C1—N1—H1A	125.5
C6—C7—C9	122.82 (19)	C8—N1—H1A	125.5

### Hydrogen-bond geometry (Å, °)

**Table:**

*D*—H···*A*	*D*—H	H···*A*	*D*···*A*	*D*—H···*A*
N1—H1A···N2^i^	0.86	2.24	3.022 (2)	151

Symmetry codes: (i) *x*, −*y*+3/2, *z*−1/2.
